# Folate content in faba beans (*Vicia faba* L.)—effects of cultivar, maturity stage, industrial processing, and bioprocessing

**DOI:** 10.1002/fsn3.192

**Published:** 2015-01-01

**Authors:** Mohammed E Hefni, Mohamed T Shalaby, Cornelia M Witthöft

**Affiliations:** 1 Food Industries Department, Faculty of Agriculture, Mansoura UniversityMansoura, Egypt; 2 Department of Food Science, Uppsala BioCenter, Swedish University of Agricultural SciencesUppsala, Sweden

**Keywords:** Bioprocessing, cultivar, faba beans, folate, industrial processing

## Abstract

Faba beans are an important source of folate and commonly consumed in Egypt. This study examined the effects of Egyptian industrial food processing (e.g., canning and freezing), germination, cultivar, and maturity stages on folate content, with the aim to develop a candidate functional canned faba bean food with increased folate content. The folate content in four cultivars of green faba beans ranged from 110 to 130 *μ*g 100 g^−1^ fresh weight (535–620 *μ*g 100 g^−1^ dry matter [DM]), which was four- to sixfold higher than in dried seeds. Industrial canning of dried seeds resulted in significant folate losses of ∼20% (*P *=* *0.004), while industrial freezing had no effect. Germination of faba beans increased the folate content by >40% (*P *<* *0.0001). A novel industrial canning process involving pregermination of dried faba beans resulted in a net folate content of 194 *μ*g 100 g^−1^ DM, which is 52% more than in conventional canned beans. The consumption of green faba beans should be recommended, providing ∼120 *μ*g dietary folate equivalents per 100 g/portion.

## Introduction

Folates are essential cofactors in one-carbon transfer reactions as donors and acceptors and are thus involved in the synthesis of purines, pyrimidines, and amino acids (Krumdieck 1990). For many populations, a folate intake below recommendation is reported (Scott et al. 2000; Dhonukshe-Rutten et al. 2007; Blancquaert et al. 2010). Insufficient dietary folate or low folate status have been suggested as possible risk factor for the occurrence of megaloblastic anemia and neural tube defects such as spina bifida and anencephaly (Blancquaert et al. 2010).

Legumes are recognized as important food sources of folate (Hoppner and Lampi 1993; Hefni et al. 2010). Legumes play an important role in the traditional diet in several regions of the world (Messina 1999). In Egypt, faba beans or broad beans (*Vicia faba* L.) are commonly consumed as a bean stew and, after germination and boiling, as a soup called *nabet*. The folate content of these traditional Egyptian foods and retention were quantified (Hefni and Witthöft 2014). The beans are harvested either in the green stage or after field-drying on the plant. The dried faba beans are commonly canned, while green faba beans are industrially frozen. No data are available on folate content in both the field-dried and the green faba beans and with respect to variation between cultivars. Data are also lacking regarding effects from industrial processing techniques, with the exception of a pilot trial in a Swedish factory which showed that mild canning of faba beans (including soaking, blanching, and retorting) did not significantly affect the folate content (Hefni and Witthöft 2014). A few studies have examined folate retention during canning of green beans, but the results are not directly comparable (Jiratanan and Liu 2004; Delchier et al. 2013).

Egypt launched a mandatory folic acid and iron fortification program for flour in August 2009 (GAIN 2009) as micronutrient deficiency is widespread in the Egyptian population. Fortification was suspended in January 2011 for technical reasons, but the aim is to resume it in future. However, alternative strategies for increasing folate intake by the Egyptian population are required in the interim. One such strategy could be to supply staple foods with increased natural folate content by bioprocessing techniques, for example, germination. During germination, the folate content is increased because of accelerated de novo synthesis in the growing seedling (Jabrin et al. 2003). Germination has been reported to increase the folate content in cereal (Jägerstad et al. 2005; Kariluoto et al. 2006; Koehler et al. 2007; Hefni and Witthöft 2011, 2012) and legume foods (Shohag et al. 2012; Hefni and Witthöft 2014). The aims of the present study were (1) to determine the effects of cultivar and maturity stage at harvesting on the folate content in common Egyptian faba bean varieties, (2) to determine the effects of soaking, canning, and freezing under authentic industrial conditions on the folate content in faba beans, and (3) to develop a candidate functional faba bean food with increased folate content using the traditional Egyptian household technique of germination combined with industrial processing.

## Materials and Methods

### Food samples and processing

#### Conventional industrial canning of dried faba beans

**Figure 1:**
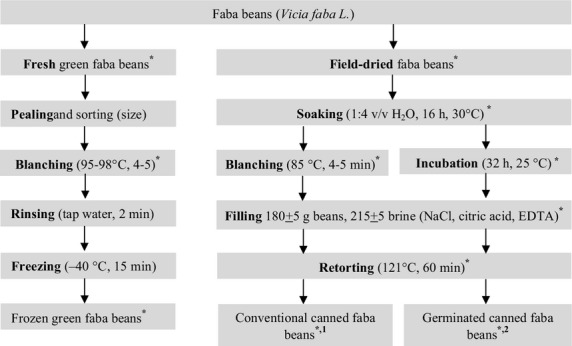
Industrial processing of green and field-dried faba beans. *Sampling points: tins of canned faba beans were opened and drained; subsamples of the drained beans and the canning medium were stored separately. ^1^The weight of drained beans was 290 ± 5 g and that of canning medium 100 ± 5 g. ^2^The weight of drained beans was 260 ± 5 g and that of the canning medium 130 ± 5 g. Samples (150 g of drained faba and 100 g of canning medium) were vacuum-packed in polyethylene bags and were transported under cooling to the Food Industries Department, Mansoura University, Mansoura, Egypt. Immediately upon arrival, samples were stored at −20°C and folate quantification was performed within 1 month.

Samples of raw (unknown imported varieties) and processed canned faba beans were provided by the Harvest Foods Company (6th of October City, Giza, Egypt). Two trials of conventional industrial canning of faba beans were carried out as outlined in Figure1. Duplicate samples (250 g each) were taken from the raw material after soaking and blanching in two independent trials. A random sample of 8–10 tins per trial of the final canned product was also collected. Immediately on arrival, the tins of canned faba bean were opened and drained. Subsamples of drained faba beans (∼150 g) and canning medium (100 g) were collected.

#### Novel industrial canning with pregerminated faba beans

In the novel industrial canning method, dried faba beans (unknown imported varieties) were first germinated in duplicate batches in a pilot plant at the Harvest Foods Company as outlined in Figure1. Total germination time (including soaking and incubation) was 48 h. Canning of the germinated faba beans was performed with minor modification of the conventional procedure by excluding blanching, while filling and autoclaving were carried under conventional conditions (Fig.1). Duplicate samples of raw material (250 g each) were collected for analysis after germination and after autoclaving. The tins of canned faba beans were opened and drained. Subsamples of the drained beans (∼150 g) and the canning medium (∼100 g) were stored at −20°C before folate quantification.

#### Effects of soaking temperature on folate content in faba beans

The effects of soaking temperature prior to canning on the folate content in faba beans were studied on pilot scale at the Food Industries Department, Mansoura University. Soaking was performed on duplicate batches of beans in a leavening cupboard (Binder, Germany) as follows. Dried faba beans (2 kg, unknown variety provided by Harvest Foods Company) were cleaned of debris and soaked in tap water (1:4 w/v) for 16 h at 20, 30, or 40°C. The water was then discarded and the soaked legumes were rinsed with fresh tap water. Subsamples (150 g) were drained, vacuum-packed in polyethylene bags, and stored at −20°C until folate quantification (within 1 month).

#### Industrial freezing of green faba beans

Samples of frozen green faba beans (Giza 641, a variety of large seeds) were provided by Nile Agricultural Ind. Co. (AGA) (Aga, Dakahlia, Egypt). Industrial freezing of green faba beans was carried out as outlined in Figure1. Duplicate samples of the raw, blanched, and frozen material were collected for analysis in two independent trials (Fig.1).

#### Effects of maturity stage and cultivar on folate content

To study the effects of maturity stage (green or dried) and cultivar on folate content, samples of green and dried beans from four cultivars of *V. faba* L. (Sakha 1, Sakha 2, Sakha 3, and Sakha 843) grown in Sakha, Kafr El-Sheikh, Egypt, in 2011, were obtained directly after harvest from Sakha Agriculture Research Station (Kafr El-Sheikh, Egypt).

Samples were vacuum-packed in polyethylene bags and stored at −20°C for folate quantification within 1 month.

### Food analysis

#### Chemicals and reagents

All chemicals were purchased from Sigma-Aldrich (St. Louis, MO) unless otherwise stated. All reagents were of pro analysis grade except acetonitrile and methanol, which were of HPLC (high-performance liquid chromatography) grade. Water was purified using a Human Power System (Seoul, Korea). Certified reference material CRM 485 (mixed vegetables) was obtained from the Institute for Reference Material and Measurement (Geel, Belgium), and subsamples were stored vacuum-packed in polyethylene bags at −20°C until analysis. The folate standards (6,S)-5,6,7,8-tetrahydrofolate sodium salt (H_4_folate), (6,S)-5-formyl-5,6,7,8-tetrahydrofolate sodium salt (5-HCO–H_4_folate), (6,S)-5-methyl-5,6,7,8-tetrahydrofolate sodium salt (5-CH_3_–H_4_folate), and pteroyl-l-glutamic acid (PteGlu) were a kind gift from Merck & Cie (Schaffhausen, Switzerland). 10-formyl folic acid sodium salt (10-HCO-PteGlu) was obtained from Dr. Schirck's laboratories (Jona, Switzerland). Folate standards were stored at −20°C until use. Standard stock solutions (∼200 *μ*g mL^−1^) were prepared according to Yazynina et al. (2008), stored under nitrogen atmosphere at −20°C, and used within 3 months. Thermostable *α*-amylase suspension (E-Blaam, Megazyme International, Wicklow, Ireland) (3000 U mL^−1^) was used during sample extraction without additional preparation. Protease suspension (5 mg mL^−1^) (Cat. No. P5147, Sigma Chemical Co., St. Louis, MO) was prepared according to Yazynina et al. (2008). The dialysed protease suspension was kept at −20°C for a maximum of 1 month. Rat serum (Agriculture Research Center, Cairo, Egypt) was dialysed (Patring et al. 2005) and stored portioned at −20°C.

#### Sample pretreatment

Before extraction, solid samples were minced frozen using a household food processor (Braun, Germany). Fluid samples were thawed overnight in the fridge. Folate quantification was performed in duplicate using trienzyme treatment (Hefni et al. 2010). In brief, 2–3 g of the food were extracted (12 min, boiling water bath) in 15 mL phosphate buffer (0.1 mol/L, pH 6.0) (containing 2% sodium ascorbate and 0.1% 2,3-dimercapto-1-propanol) with addition of thermostable *α*-amylase (60 *μ*L). The samples were then cooled on ice and treated with protease suspension (0.8 mL protease, 37°C, 90 min). The extract obtained was heated for 5 min in a boiling water bath and centrifuged. Folate polyglutamates in the samples were deconjugated by addition of dialysed rat serum (200 *μ*L mL^−1^ sample extract) and incubation at 37°C for 2 h. Purification of extracts was carried out by solid phase extraction using strong anion exchange cartridges (500 mg, Isolute, Hypersep, Thermo Scientific, Waltham, MA, USA) as described by Hefni et al. (2010). The preconditioned cartridges were loaded with 2.5 mL sample extract, washed, and folate was eluted with 4 mL elution buffer containing 0.1% 2,3-dimercapto-1-propanol.

#### Folate quantification by HPLC

Folates were quantified using RP-HPLC-UV/FLD (Shimadzu LC10, Kyoto, Japan). Folates were separated on an Aquasil C_18_ column (3 *μ*m, 150 × 4.6 mm, Thermo Scientific). An external multilevel (*n* = 8) calibration curve was used for quantification with minor modification from earlier published procedure (Hefni et al. 2010). Quantification was based on fluorescence detection (ex/em 290/360 nm for reduced folates and 360/460 nm for 10-HCO–PteGlu). 5-HCO–H_4_folate was quantified by UV detection (290 nm) for greater sensitivity and dual detection (UV/FLD) was used to confirm peak identity and purity.

#### Quality control of the analytical method

The calibration curves of different folate forms were linear from 0.4 to 80 ng mL^−1^ for H_4_folate, 0.3 to 110 ng mL^−1^ for 5-CH_3_–H_4_folate, 1 to 200 ng mL^−1^ for 10-HCO–PteGlu, 4 to 200 ng mL^−1^ for 5-HCO–H_4_folate, and 4 to 100 ng mL^−1^ for PteGlu. The lowest correlation coefficient (*R*^2^ = 0.998) was found to 5-HCO–H_4_folate, all others were above (*R*^2^ = 0.999). Recovery tests were carried out by addition of folate standards at two concentrations (50% and 100% of the initial folate content) before extraction to both green and field-dried faba beans. As in-house control sample, dried faba beans (50 g) were placed in screw capped tubes, flushed with nitrogen, stored at −20°C, and milled prior to extraction. Certified reference material CRM 485 was used for method validation (analyzed in duplicate). Inter- and intra-assay variability was determined by variation coefficients of analytical replicates (*n* = 6).

#### Dry matter determination

Dry matter was determined in duplicate on all solid samples directly before folate extraction according to AOAC (2000).

### Calculations and statistics

Mean folate content (sum of individual folate forms) was expressed as *μ*g folic acid 100 g^−1^ food dry matter (mean ± SD, *n* = 2, duplicate trials and analyses) after conversion using a molecular weight of 445.4 for H_4_folate, 459.5 for 5-CH_3_–H_4_folate, 469.4 for 10-HCO–PteGlu, and 473.5 for 5-HCO–H_4_folate. Folate content per food portion was expressed in Dietary Folate Equivalents (DFE) (Suitor and Bailey 2000; Yang et al. 2005). A general linear model was used to analyze the effects of the treatments on folate content. Differences between raw and processed materials at each step were compared using Tukey's pairwise comparison, with the level of significance set at *P *<* *0.05. All statistical analyses were carried out using SAS software Version 9.1 (SAS Institute Inc., Cary, NC).

## Results

The folate forms H_4_folate, 5-CH_3_–H_4_folate, 10-HCO–PteGlu, and 5-HCO–H_4_folate were quantified in raw as well as processed dried faba beans. In green faba beans, 10-HCO–PteGlu was not detected (Table1). The repeatability (intra- and interassay) of the analytical procedure was evaluated by analyzing dried faba beans (as in-house control sample). The intra- and interassay was below 12% (*n* = 6) and 8% (*n* = 3), respectively, for all folate forms. These results were consistent with data from others (Kariluoto et al. 2006) who reported a variation between duplicate analyses below 10% except for some autoclaved and puffed germinated rye samples (16% and 17%).

**Table 1:** Folate content (*μ*g ± SD 100 g^−1^ DM) in the four faba bean (*Vicia faba* L.) cultivars harvested at the green and field-dried stage

	Green faba beans						Field-dried faba beans					
Variety	DM	H_4_folate	5-CH_3_–H_4_folate	10-HCO–PteGlu	5-HCO–H_4_folate	Sum as PteGlu ± SD	DM	H_4_Folate	5-CH_3_–H_4_folate	10-HCO–PteGlu	5-HCO–H_4_folate	Sum as PteGlu ± SD
Sakha 1	21 ± 1	29 ± 0.2	493 ± 56.2	ND	35 ± 4.3	535 ± 57.8^A^	90 ± 1	10 ± 3.8	38 ± 2.2	13 ± 2.2	36 ± 0.9	92 ± 7.3^C,B^
Sakha 2	21 ± 1	27 ± 2.1	556 ± 1.4	ND	64 ± 4.1	621 ± 5.6^A^	89 ± 1	8 ± 0.6	49 ± 5.7	15 ± 1.4	18 ± 3.0	86 ± 10.3^B^
Sakha 3	21 ± 1	33 ± 1.6	537 ± 37.8	ND	69 ± 2.8	614 ± 31.8^A^	90 ± 1	11 ± 0.8	65 ± 8.7	10 ± 1.4	31 ± 3.4	111 ± 12^C,B^
Sakha 843	23 ± 1	16 ± 0.9	491 ± 50.7	ND	69 ± 7.5	552 ± 54.8^A^	90 ± 1	24 ± 3.0	62 ± 8.9	12 ± 2.5	49 ± 2.5	140 ± 16.1^C^

Individual folate values are means of duplicate samples and duplicate analyses (*n* = 2). The sum of folates was calculated from individual folate forms using the conversion factors 445.4 for tetrahydrofolate (H_4_folate), 459.5 for 5-methyl-tetrahydrofolate (5-CH_3_–H_4_folate), 469.4 for 10-formyl folic acid (10-HCO–PteGlu), and 473.5 for 5-formyl-tetrahydrofolate (5-HCO–H_4_folate). No significant differences were observed for folate content in varieties of fresh green faba beans (*P *=* *0.05). Different superscripts within the same column represent significant differences (*P *<* *0.05). DM, dry matter; ND, not detected.

The variation in folate content between the two independent germination and canning trials was below 10% (data not shown). In CRM 485, the folate forms H_4_folate (5 ± 1 *μ*g 100 g^−1^) and 5-CH_3_–H_4_folate (201 ± 3 *μ*g 100 g^−1^) were quantified. These results are in agreement with an indicative value for 5-CH_3_–H_4_folate of 214 ± 42 *μ*g 100 g^−1^ (Finglas et al. 1999). However, the sum of the individual folate vitamers expressed as folic acid (204 *μ*g 100 g^−1^) was lower than the certified total folate content (315 ± 28 *μ*g 100 g^−1^) determined by microbiological assay (Finglas et al. 1999). This result is consistent with the expected discrepancy between these methods (Koontz et al. 2005; Puwastien et al. 2005; Phillips et al. 2010). Relative recovery of individual folate forms (after addition of 50% and 100% of the initial folate content before extraction) in both green and field-dried faba beans (*n* = 8) was 88 ± 7% for H_4_folate, 92 ± 8% for 5-CH_3_–H_4_folate, 90 ± 9% 10-HCO–PteGlu, and 78 ± 7% for 5-HCO–H_4_folate.

Mean folate content in the green faba beans cultivars included in the study ranged from 110 to 130 *μ*g 100 g^−1^ fresh weight or 535 to 620 *μ*g 100 g^−1^ dry matter (DM), which did not vary significantly (*P *=* *0.326) and was approximately four- to sixfold higher than in the dried beans (Table1). The highest folate content (140 *μ*g 100 g^−1^ DM, *P *=* *0.0357) in dried faba beans was found in cultivar Sakha 843. The folate vitamer composition differed between green and dried beans. 5-CH_3_–H_4_folate was the major vitamer present, comprising up to 90% of total folate in the green faba beans and up to 60% in the dried beans (Table1). 10-HCO–PteGlu was not detected in green faba beans, while in dried beans it comprised up to 17% of total folates (Table1).

Conventional industrial canning of dried beans resulted in net folate losses of ∼20% (*P *=* *0.004), with an initial increase after soaking and successive losses after blanching and autoclaving (Table2). A soaking temperature (prior to canning) of 20 or 30°C resulted in a similar significant increase in folate content (25% compared with raw/untreated beans, *P = *0.0002), while soaking at 40°C did not increase the folate content (*P *=* *0.935) compared with raw beans (Table3).

**Table 2:** Folate content (*μ*g ± SD 100 g^−1^) in dried faba beans during conventional industrial canning

	Fresh weight						Dry weight					
Processing step	DM	H_4_folate	5-CH_3_–H_4_folate	10-HCO–PteGlu	5-HCO–H_4_folate	Sum as PteGlu ± SD	H_4_Folate	5-CH_3_–H_4_folate	10-HCO–PteGlu	5-HCO–H_4_-folate	Sum as PteGlu ± SD	% relative content as PteGlu (DM)^1^
Raw	91 ± 0.1	18 ± 0.5	61 ± 6.4	10 ± 0.6	61 ± 4.1	142 ± 10	20 ± 0.6	67 ± 7.1	11 ± 0.7	67 ± 4.5	157 ± 6.9^B^	100
Soaked	47 ± 1.1	10 ± 0.4	44 ± 1.7	2 ± 0.1	32 ± 2.7	85 ± 4.1	21 ± 0.9	95 ± 3.6	5 ± 0.2	70 ± 5.7	181 ± 8.8^A^	115
Blanched	44 ± 0.3	5 ± 0.2	45 ± 2.3	3 ± 0.3	27 ± 2.9	76 ± 0.5	10 ± 0.5	103 ± 3.7	6 ± 0.8	60 ± 3.8	171 ± 1.2^A,B^	109
Autoclaved	27 ± 0.2	3 ± 0.6	18 ± 1.6	1 ± 0.1	14 ± 2.2	35 ± 3.5	12 ± 2.1	68 ± 6.0	5 ± 0.5	51 ± 8.2	129 ± 12.8^C^	81
Canning medium^2^		2 ± 0.2	14 ± 1.7	3 ± 0.4	3 ± 0.9	21 ± 1.5						

Individual folate values are means of duplicate trials and duplicate analyses (*n* = 2). The sum of folates was calculated from individual folate forms using the conversion factors 445.4 for tetrahydrofolate (H_4_folate), 459.5 for 5-methyl-tetrahydrofolate (5-CH_3_–H_4_folate), 469.4 for 10-formyl folic acid (10-HCO–PteGlu), and 473.5 for 5-formyl-tetrahydrofolate (5-HCO–H_4_folate). Different superscripts within the same column represent significant differences (*P *<* *0.05). DM, dry matter.

1 The relative content was calculated for the sum of PteGlu on dry weight basis.

2 Drained weight of beans was 290 ± 5 g and of canning medium 100 ± 5 g.

**Table 3:** Folate content (*μ*g ± SD 100 g^−1^ DM) in dried faba beans after soaking at different temperatures for 16 h

		Folate vitamer (*μ*g 100 g^−1^ DM)				
Soaking temperature (°C)	DM (g 100 g^−1^)	H_4_folate	5-CH_3_–H_4_folate	10-HCO–PteGlu	5-HCO–H_4_folate	Sum as PteGlu ± SD
Untreated	91 ± 0.1	20 ± 0.6	67 ± 7.1	11 ± 0.7	67 ± 4.5	157 ± 6.9^B^
20 ± 2	47 ± 0.6	23 ± 3.4	129 ± 9.5	4 ± 0.5	54 ± 3.1	202 ± 11.7^A^
30 ± 2	46 ± 0.5	19 ± 2.9	142 ± 11.1	3 ± 0.6	58 ± 4.8	212 ± 12.5^A^
40 ± 2	43 ± 0.8	8 ± 0.6	108 ± 8.4	4 ± 0.2	50 ± 4.8	162 ± 11.6^B^

Individual folate values are means of duplicate analyses from duplicate trials (*n* = 2). The sum of folate was calculated from individual folate forms using the conversion factors 445.4 for tetrahydrofolate (H_4_folate), 459.5 for 5-methyl-tetrahydrofolate (5-CH_3_–H_4_folate), 469.4 for 10-formyl folic acid (10-HCO–PteGlu) and 473.5 for 5-formyl-tetrahydrofolate (5-HCO–H_4_folate). Different superscripts within the same column represent significant differences (*P *<* *0.05). DM, dry matter.

Blanching, freezing, and subsequent storage (6 months at −20°C) of green faba beans did not significantly affect the folate content (Table4).

**Table 4:** Folate content (*μ*g ± SD 100 g^−1^) in green faba beans during industrial freezing

	Fresh weight					Dry weight				
Processing step	DM	H_4_folate	5-CH_3_–H_4_folate	5-HCO–H_4_folate	Sum as PteGlu ± SD	H_4_folate	5-CH_3_–H_4_folate	5-HCO–H_4_folate	Sum as PteGlu ± SD	% relative content as PteGlu^1^
Raw	29 ± 1.8	7 ± 0.7	97 ± 6.2	6 ± 1.6	106 ± 7.7	23 ± 2.3	334 ± 21	22 ± 5.6	364 ± 27	100
Blanched	25 ± 0.7	6 ± 0.8	83 ± 6.8	2 ± 1.0	88 ± 6.8	22 ± 4.1	333 ± 27	9 ± 4.5	350 ± 27	96
Frozen	25 ± 2.9	5 ± 0.9	84 ± 3.6	2 ± 0.2	88 ± 3.5	21 ± 1.6	338 ± 44	8.5 ± 1.2	353 ± 41	97
Frozen (6 months at −20°C)	25 ± 3.5	5 ± 0.9	88 ± 4.3	2 ± 0.3	92 ± 3.3	21 ± 7.8	355 ± 57	9.2 ± 0.9	370 ± 53	101

Individual folate values are means of duplicate trials and duplicate analyses (*n* = 2). The sum of folates was calculated from individual folate forms using the conversion factors 445.4 for tetrahydrofolate (H_4_folate), 459.5 for 5-methyl-tetrahydrofolate (5-CH_3_–H_4_folate) and 473.5 for 5-formyl-tetrahydrofolate (5-HCO–H_4_folate). 10-HCO–PteGlu was not detected. No significant differences (*P *>* *0.05) were observed for folate content during processing as compared to the raw green faba beans (dry matter basis). DM, dry matter.

1 The relative content was calculated for the sum of PteGlu on dry weight basis.

The novel industrial canning process for dried faba beans (canning after germination) resulted in a net folate increase in the final product of ∼30% (Table5). Germination, including soaking and subsequent incubation (∼25°C, 48 h) resulted in a >40% higher folate content (based on DM) compared with the raw material, mainly due to an increase in 5-CH_3_–H_4_folate content (Table5). No increase in 10-HCO–PteGlu and 5-HCO–H_4_folate content was observed (Table5). The folate content in the novel germinated–canned faba beans was 194 *μ*g 100 g^−1^ DM, which was 52% higher than in the conventional canned faba bean product, based on DM.

**Table 5:** Folate content (*μ*g ± SD 100 g^−1^) in dried faba beans (48 h, 25°C) during the novel industrial canning process with a pregermination step

	Fresh weight						Dry weight					
Processing step	DM	H_4_folate	5-CH_3_–H_4_folate	10-HCO–PteGlu	5-HCO–H_4_folate	Sum as PteGlu	H_4_folate	5-CH_3_–H_4_folate	10-HCO–PteGlu	5-HCO–H_4_folate	Sum as PteGlu	% relative content as PteGlu^1^
Raw	90 ± 1.0	18 ± 0.5	61 ± 6.4	10 ± 0.6	61 ± 4.1	142 ± 10	20 ± 0.6	67 ± 7.1	11 ± 0.7	67 ± 4.5	157 ± 6.9^C^	100%
Germinated	38 ± 0.1	10 ± 0.9	45 ± 2.4	9 ± 2.8	23 ± 2.03	84 ± 7.9	28 ± 2.6	120 ± 6.6	24 ± 7.6	62 ± 5.4	223 ± 21^A^	142%
Autoclaved	21 ± 0.1	5 ± 0.4	14 ± 0.5	5 ± 0.3	18 ± 0.4	40 ± 1.2	25 ± 2.1	67 ± 2.5	25 ± 1.5	87 ± 1.5	194 ± 3.7^B^	124%
Canning medium^2^		2 ± 0.2	17 ± 1.7	3 ± 0.4	4 ± 0.9	25 ± 1.5						

Individual folate values are means of duplicate samples and duplicate analyses (*n* = 2). The sum of folates was calculated from individual folate forms using the conversion factors 445.4 for tetrahydrofolate (H_4_folate), 459.5 for 5-methyl-tetrahydrofolate (5-CH_3_–H_4_folate), 469.4 for 10-formyl folic acid (10-HCO–PteGlu) and 473.5 for 5-formyl-tetrahydrofolate (5-HCO–H_4_folate). Different superscripts within the same column represent significant differences (*P *<* *0.05) on a dry matter basis. DM, dry matter.

1 The relative content was calculated for the sum of PteGlu on dry weight basis.

2 Drained weight of beans was 290 ± 5 g and of canning medium 100 ± 5 g.

## Discussion

The high folate content in faba beans and the high consumption of faba bean products in Egypt (Bakr and Bayomy 1997) means that faba bean products are important folate sources in the Egyptian diet. Hence, information regarding the effects of cultivars and maturity stage on folate content could be helpful to improve dietary intake. This study confirmed that fresh green faba beans are a rich source of folate (>100 *μ*g 100 g^−1^ fresh weight) and showed that further industrial processing, including blanching, freezing, and storage (up to 6 months at −20°C), did not significantly affect the folate content. Both fresh and frozen green faba beans are thus recommended for consumption. A portion size of 100 g green faba beans would provide 120 *μ*g DFE.

However, the folate content in field-dried faba beans was significantly affected by cultivar and maturity stage. Similar findings have been reported for other food crops, with folate content significantly affected by cereal cultivar (e.g., wheat) (Piironen et al. 2008; Hefni and Witthöft 2012) and maturity stage of some vegetables (e.g., tomato and strawberries) (Strålsjö et al. 2003; Periago et al. 2008; Iniesta et al. 2009). Periago et al. (2008) reported a >50% decrease in folate content in tomatoes on maturing from green to red stage. Similarly, we found that the folate content (based on DM) in faba beans decreased by >70% from green to dried stage. Harvesting the beans at the field-dried stage also altered the relative distribution of individual folate forms, with the 10-HCO–PteGlu content increasing from 0% (in green faba beans) to 17% (in dried beans) (Table1). This is possibly due to interconversion and/or oxidation, as found by others (Pfeiffer et al. 1997; De Brouwer et al. 2007). Pfeiffer et al. (1997) reported rapid oxidation of 10-HCO–H_4_folate via 10-HCO–H_2_folate to 10-HCO–PteGlu. 5-HCO–H_4_folate can also be converted via 5,10-CH–H4folate to 10-HCO–PteGlu (Pfeiffer et al. 1997; De Brouwer et al. 2007).

The high retention of folate during canning of field-dried faba beans in this study (>80%) and in other trials (Hefni and Witthöft 2014) confirms that conventional canned faba bean products are a good folate source. However, the present study also showed that the folate content of canned faba beans can be increased by including germination in the process. In a previous pilot trial, we observed a >70% increase in folate content during soaking and subsequent incubation of faba beans (48 h) (Hefni and Witthöft 2014). Shohag et al. (2012) reported similar findings, with an up to 3.9-fold increase in the folate content of germinated soybeans and mung beans. The increase of the folate content during soaking is probably due to enzymatic de novo synthesis from initiated germination (Jabrin et al. 2003). In the present study, the conventional canning process for faba beans was modified by including a germination step, which resulted in a 52% higher folate content in the novel product compared to the conventional. A serving of 150 g (including 30% canning medium, which is commonly consumed in Egypt) of this novel product would supply 65 *μ*g DFE and can be recommended as a candidate functional food with increased folate content (compared with 40 *μ*g in the conventional product).

Data showed that both germinated–canned faba beans and green faba beans are good folate sources. However, green faba beans supply more folate per portion, whereas storage of canned faba beans is easier and does not require a freezer. Both could be recommended for regular consumption.

In recent years, advances in the determination of the folate content of foodstuffs were made using mass spectrometric detection (stable isotope dilution) (Phillips et al. 2006; Patring and Jastrebova 2007; Ringling and Rychlik 2013). These methods require relatively expensive instrumentation that is not universally available. The current study was carried out using standard HPLC equipment with ultraviolet spectrometric and fluorescence detection (HPLC-UV/FLD). However, a limitation is the difficulty to quantify 5-HCO–H_4_folate which has a low fluorescence response. Also others (Ruggeri et al. 1999; Gujska and Kuncewicz 2005; Kariluoto et al. 2006) reported difficulties when quantifying 5-HCO–H_4_folate using HPLC. Therefore, in the current study, 5-HCO–H_4_folate was quantified by UV detection for greater sensitivity and dual detection (peak ratio UV/FLD) was used to confirm peak identity and purity. Thorough method validation was performed to achieve reliable new data.

## Conclusions

Maturity stage, cultivar, and industrial canning affected the folate content of field-dried faba beans significantly, while industrial freezing had no effect on green faba beans. Therefore, green faba beans can be recommended as good folate source. The novel industrial canning process for dried faba bean seeds, which included a novel germination step, resulted in a net folate increase of >50%. Canned germinated faba beans are thus a candidate functional product with increased folate content.
